# System architecture and interactive LLM prompt framework for the integration of clinical and mitochondrial data in obesity: a software prototype and feasibility study

**DOI:** 10.3389/frai.2026.1923426

**Published:** 2026-09-10

**Authors:** Karenth Milena Rodríguez-Córdoba, William Darío Ávila-Díaz

**Affiliations:** 1 University of Guadalajara, Guadalajara, Mexico; 2 Clinica Sanitas Sebastian de Belalcazar, Cali, Colombia; 3 Postdoctoral University of Guadalajara, Guadalajara, Mexico

**Keywords:** large scale language models (LLM), local storage (IndexedDB), mitochondria, obesity, prompt engineering, translational medicine

## Abstract

**Introduction:**

Obesity is a critical global health challenge due to its close association with cardiovascular and metabolic risk. At the molecular level, the lipotoxic environment alters the structure, dynamics, and connectivity of the mitochondrial network. Despite advances in cell biology and digital health, there is a lack of lightweight computational tools capable of integrating clinical records with molecular and photomicrographic data in a standardized way. This study presents the design, development, and feasibility assessment of a decoupled software prototype and an interactive prompt engineering framework for integrating clinical and histological mitochondrial data in molecular obesity research.

**Methodology:**

A client-side software architecture was designed using HTML5, CSS3, and the IndexedDB API for the transactional and anonymized persistence of medical records (coded using the ICD-10 classification) and micrographs of human placental tissue (obtained using transmission electron microscopy, immunofluorescence, and immunohistochemistry for the evaluation of mitochondrial fusion molecular markers such as Mitofusin-2, nitrotyrosine). For interpretive processing, a framework of prompts structured under the Chain-of-Thought paradigm was built, designed to interact with Large-Scale Language Models (LLMs). The interface’s usability, local storage performance, and the consistency of AI-assisted reasoning were evaluated.

**Results:**

The developed platform enabled the seamless and instantaneous management of independent patient tabs (unique storage keys mitochondria_001 to 010), achieving local read/write latency without reliance on external servers or transmission of sensitive personal data. The prompt framework proved effective in guiding LLMs in translating mitochondrial biomarkers and clinical data into structured summaries of oxidative stress status and metabolic risk. The lack of a trained computer vision model for automatic segmentation is declared as the main limitation of the current study.

**Conclusion:**

The software prototype and prompt interface provide a low-cost, secure (data privacy-oriented), and clinically intuitive solution for translational data management in obesity. This work demonstrates the feasibility of integrating heterogeneous biomedical parameters using conversational AI tools and local storage, laying the groundwork for the future incorporation of image segmentation algorithms.

## 1 Introduction

Obesity represents one of the most complex and impactful health challenges in global public health, constituting a primary risk factor for cardiovascular and endocrine-metabolic morbidity and mortality (Ezzati, 2004; World Health Organization, 2002, 2017, 2024; Food and Agriculture Organization of the United Nations, 2016, 2023; World Obesity Federation, 2025). At the population and regional levels, the sustained increase in the prevalence of this condition and non-communicable diseases demands a comprehensive approach from the gestational and pediatric stages to adulthood (Boney et al., 2005; ENSIN/Ministry of Health and Social Protection of Colombia, 2015; Fonseca et al., 2010; Fonseca et al., 2011; Global Observatory for Physical Activity, 2024).

At the cellular and placental level, the excess of nutrients and the lipotoxic environment characteristic of this pathology alter the physiological mechanisms of intermediate metabolism (Bax and Bloxam, 1997; Mandò et al., 2018; Dudek, 2020), favoring the development of insulin resistance and directly impacting the bioenergetics, morphology, turnover and connectivity of mitochondrial networks (Kelly et al., 2020; Fowden et al., 2021; Cindrova -Davies and Sferruzzi -Perri, 2022; Reynolds et al., 2013). Mitochondria, as central organelles in the control of oxidative stress, fetal development and signaling against DNA damage, undergo structural modifications and accumulation of reactive oxygen species (ROS) (Shimura, 2023; Rodríguez et al., 2025; Rodríguez et al., 2025) that mark the beginning and progression of translational metabolic dysfunction (Vaughan and Fowden, 2016; Fisher et al., 2021; Juan et al., 2021; Shimura, 2023).

In current biomedical research, the analysis of mitochondrial metabolism and dynamics in the contexts of obesity, gestational diabetes, and microvascular damage has advanced significantly through biological models and advanced molecular histopathology techniques (Chang et al., 2021, 2022; Hernández et al., 2022; Sun et al., 2022), such as transmission electron microscopy (TEM), immunofluorescence, and immunohistochemistry (Diceglie et al., 2021; Kolac et al., 2021). For example, Shimura (2023) demonstrated that mitophagy is compromised in obesity due to inefficient activation of the PINK1/Parkin pathway secondary to the downregulation of Mitofusin-2 (Mfn2). Consistently, Bulbul Ahmed et al. (2021) reported significant reductions in Mfn2 associated with insulin resistance, while Rodríguez et al. (2025), Córdoba and Pustovrh (2024), and Chang et al. (2022) showed how blocking mitochondrial turnover induces the accumulation of dysfunctional organelles, increasing nitrotyrosine residues and ROS production, while reducing ATP synthesis.

Despite the high biological value of these findings on nutrient transporters and trophoblastic function (James-Allan et al., 2020; Fisher et al., 2021), the routine clinical assessment of patients with obesity continues to rely predominantly on conventional anthropometric parameters (weight, height, and Body Mass Index [BMI]). A substantial gap persists in the ability to integrate this clinical data with subcellular information and mitochondrial health immunomarkers in an accessible, structured, and actionable way for daily medical practice. Although recent artificial intelligence tools and generalist multimodal medical models have begun to be applied to risk prediction and patient characterization (Jeong et al., 2023; Moor et al., 2023; De la Torre et al., 2024; Azmi et al., 2025), or specialized tissue-specific pathology algorithms such as Placenta CLIP+ (Pan et al., 2024), there is a shortage of architectures for lightweight software capable of centralizing the patient’s medical history and linking it directly with storage of tissue photomicrographs and molecular immunolabeling studies under data privacy and health equity standards (Clark et al., 2021).

In this context, the central question of this research arises:

prompt engineering framework for the integration, structuring, and contextualization of clinical data and mitochondrial immunomarkers in obesity?

To answer this question, this work was designed to develop and evaluate a client -side software prototype based on HTML5 and the IndexedDB API, combined with a pipeline of interactive prompts for Large-Scale Language Models (LLMs). This platform allows for the local, transactional, and anonymized management of medical records (coded under ICD-10) along with the preview of micrographs of human placental tissue (obtained from previous studies of gestational obesity labeled for Mfn2 and nitrotyrosine; Córdoba and Pustovrh, 2024). In turn, the Chain-of-Thought prompt framework guides the language model in the contextualized interpretation of molecular and clinical variables.

The relevance of this feasibility study lies in its translational approach: it provides a prototype of secure digital support (which preserves patient privacy by not transmitting data to external servers) and lays the computational groundwork for the future incorporation of AI segmentation algorithms. In this way, it seeks to bridge the gap between basic molecular biology and precision clinical medicine in high-risk metabolic, cardiovascular, and oncological diseases.

## 2 Methodology

### 2.1 Study design and system architecture

A technological development, proof of concept, and feasibility study (Software Prototype and Feasibility Study) was designed. Based on a comprehensive literature review, the absence of integrated, locally accessible platforms that allow direct linking of obese patient medical records with histopathological study storage was identified. Molecular micrographs of cells and subcellular micrographs. To address this gap, a decoupled client -side web platform was developed, combined with a structured prompt engineering framework designed to interact with Large Scale Language Models (LLMs) and thus facilitate patient management and their application in clinical practice, improving doctor-patient interaction times.

### 2.2 Building the local database

For the construction of the local database and the evaluation of the system, the following were included: Records of deidentified medical histories of patients diagnosed with gestational obesity and controls with normal weight, which included anthropometric variables, lipid profile, vital signs and coding of diagnoses according to the International Classification of Diseases (ICD-10). Microphotomicrographs of human placental tissue from previous biomedical research that have been approved by an ethics committee for the use of microphotographic studies for subsequent studies, containing molecular characterization by Transmission Electron Microscopy (TEM) for the evaluation of mitochondrial morphology and ultrastructure.

Immunofluorescence and Immunohistochemistry with specific labeling for the, nitrotyrosine, Mitofusin-2 (Mfn2) receptor using primary and secondary antibodies (secondary antibody conjugated with Alexa Fluor 488, nuclear staining with DAPI and cytoskeleton stained with Rhodamine-Phalloidin). Immunohistochemistry for the detection of mitochondrial fusion mitofusion-2.

### 2.3 Bibliographic search strategy

Register databases. Controlled Trials (CENTRAL), LILACS and PubMed, covering publications between 2020 and 2025. Search equations were structured adapted to each database using MeSH/Emtree descriptors and Boolean operators combining terms such as: “obesity”, “mitochondrial dynamics”, “mitofusin-2”, “reactive oxygen species”, “natural language processing”, “prompt engineering”, “large language models and health information systems”. Additionally, further sources such as Google Scholar, ClinicalTrials.gov, thesis databases and clinical practice guidelines on obesity and molecular endocrinology were consulted, without language restriction.

### 2.4 Data acquisition and development of the local web platform

#### 2.4.1 Structuring of medical records and client database (IndexedDB)

A structured data model was designed for clinical management using the IndexedDB transactional API integrated into the web browser (HTML5/JavaScript). The interface allows for the local and persistent storage of a clinical module, including the reason for consultation, current illness, vital signs, primary and secondary diagnoses (ICD-10 scale), and treatment plan. It also includes a molecular and imaging module with attached micrograph files encoded in Base64 strings, classified according to the microscopy technique used (TEM, immunofluorescence, and immunohistochemistry), and uniquely associated with the patient’s anonymized code (e.g., mitochondria_001).

#### 2.4.2 Code availability statement and supplementary material

The complete source code for the client-side web interface (HTML5, CSS3, vanilla JavaScript) and the local IndexedDB database schemas presented in this study have been relocated from the main text to focus on the scientific contributions. The fully functional source code is publicly accessible on GitHub^1^ and is provided as Supplementary Material S1.

### 2.5 Characterization of micrographs and molecular markers

The images integrated into the platform are from a previous cohort study comparing gestational obesity against normal-weight controls. The samples include immunohistochemistry for Mitofusin-2, nitrotyrosine, and assessment of the relative expression of the mitochondrial fusion receptor (brown staining) and the accumulation of oxidative/nitrosative damage. Immunofluorescence Confocal: Detection of Mfn2 (green channel, Alexa Fluor 488), delineation of the actin cytoskeleton (red channel, Rhodamine-Phalloidin) and nuclear labeling (blue channel, DAPI). Transmission Electron Microscopy (TEM): Direct ultrastructural analysis of the density of the cristae, degree of fragmentation or elongation of the network and presence of autophagic vacuoles.

### 2.6 Prompt engineering framework for LLM and contextual processing

Prompt engineering framework (LLM Prompt). Engineering Framework, designed under the Chain-of-Thought principle. This pipeline guides Large Scale Language Models in the holistic analysis and interpretation of the variables entered into the platform.

#### 2.6.1 Structure and design of interactive prompts

Prompts framework was constructed by parameterizing five fundamental analytical dimensions:

1. Advanced multimodal integration: defines the role of the system as a specialized assistant in translational medicine, ordering the joint evaluation of anthropometric data, the ICD-10 scale and the reported levels of Mfn2, ROS and mitochondrial fragmentation.
2. Comparative mapping normal vs. pathological (1:1): models the cause-and-effect relationship between the lipotoxic environment of obesity and the cellular response, evaluating morphological changes, alteration of membrane potential, accumulation of nitrotyrosine and activation of the mitophagy pathway mediated by PINK1/Parkin.
3. Contextualization of microscopy methods that provides guidelines for interpreting the findings of optical microscopy, immunofluorescence and TEM, recognizing the methodological strengths and limitations of each technique.
4. Subcellular mechanistic analysis: details the pathophysiological flows by which lipotoxicity induces endoplasmic reticulum stress and protein damage in placental tissue.
5. Synthetic output model: generates a clinical-structured summary that suggests the stratification of the patient’s metabolic risk and proposes the correlation between the observed mitochondrial health and possible clinical-nutritional interventions.

### 2.7 Feasibility, usability and technical performance assessment

The validation of the software prototype focused on two key components: Technical Performance and Latency: Measurement of processing time and local storage memory consumption (IndexedDB) during loading, retrieval and navigation between 10 medical records with attached high-resolution micrographs, which can be extended to more depending on the type of programming and new tabs can be added to create a robust database that allows you to store a large amount of personalized medical history data.

**Figure 1:**
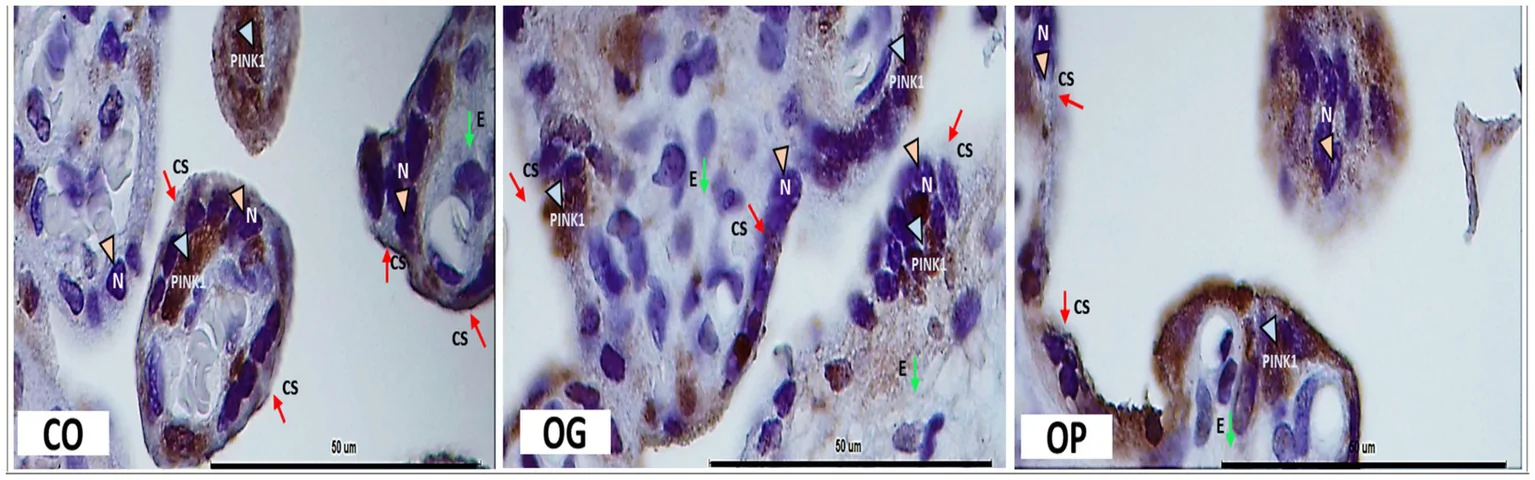
Mitofusin 2 receptor markers (brown color), immunohistochemical markers in human placental tissue of group. CO: Control, Group OG: Gestational obese, Group OP: Pre-gestational obese.

Expert-Assisted Coherence Assessment: The synthetic reports and interface usability were qualitatively assessed by two independent experts in molecular histopathology and clinical endocrinology, verifying the conceptual accuracy of the interpretations generated using the prompt template compared to conventional clinical judgment (see Figure 1).

### 2.8 Large language model specifications, configuration, and reproducibility

**Figure 2:**
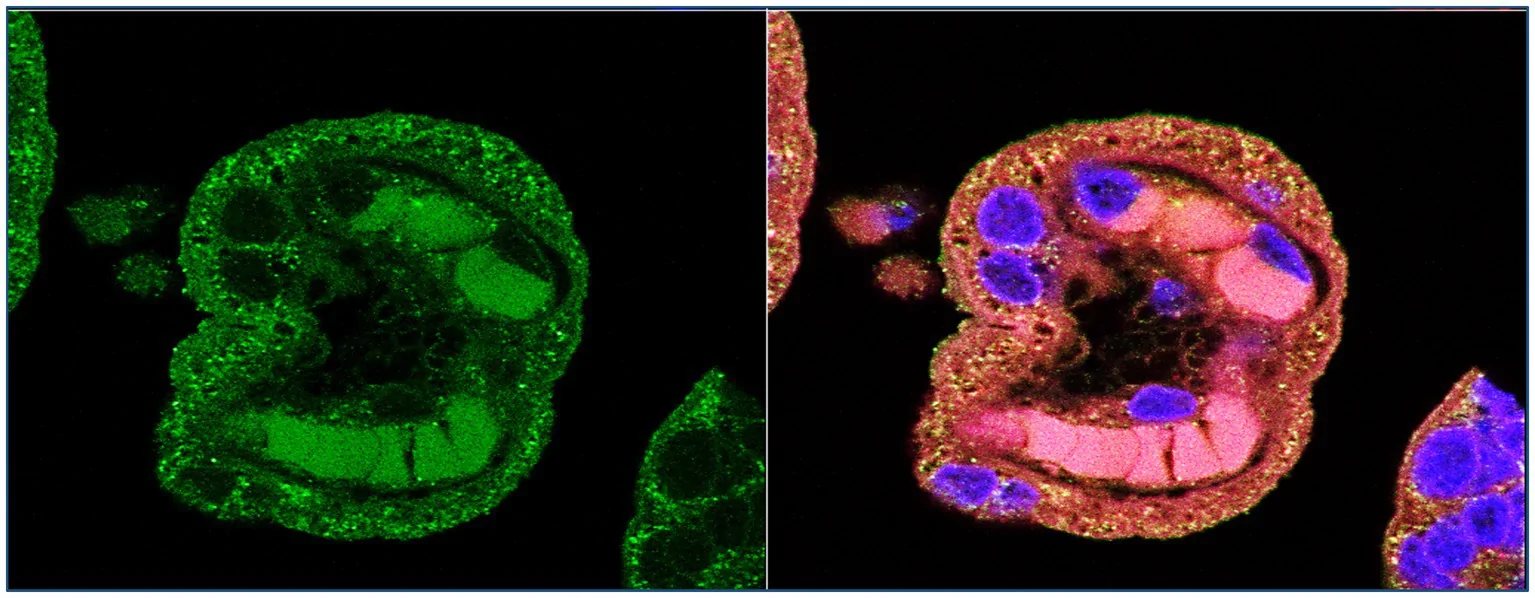
Mitofusin 2 receptor markers (green), Alexa Fluor 488 immunofluorescence markers, in human placental tissue from group. CO: Control, group OG: Gestational obese, group OP: Pre-gestational obese. Blue: DAPI, Red: Rhodamine.

**Figure 3:**
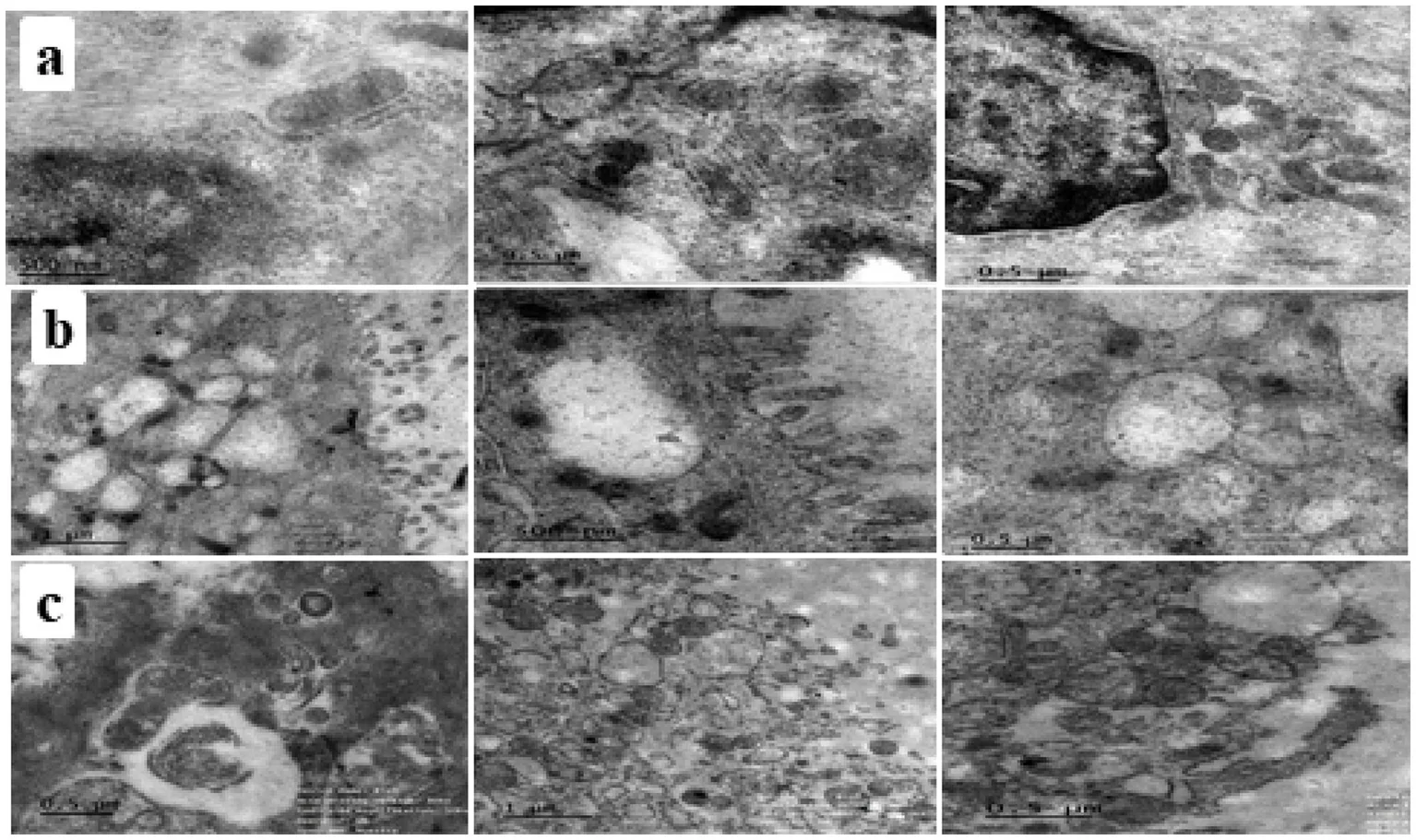
Transmission electron microscopy study, mitochondrial morphology is observed in human placental tissue of group. CO: control, group OG: gestational obese, group OP: pre-gestational obese.

To evaluate the interactive prompt framework and the generation of integrative clinical-molecular summaries, we utilized the GPT-4o architecture (version gpt-4o-2024-08-06) via API/structured interface. To mitigate output variability, prevent clinical hallucination, and ensure scientific reproducibility of the Chain-of-Thought (CoT) reasoning, model generation parameters were strictly configured as follows: Temperature ( T ): Set to 0.0 (deterministic configuration to minimize stochastic variation and guarantee logical consistency across evaluations). Top_p: Set to 1.0. Presence and Frequency Penalty: Set to 0.0. Output Variability & Reproducibility: Five ( 5 ) independent runs were executed for each of the 10 structured patient records (mitochondria_001 through 010). Under T=0.0 , syntactic and semantic concordance of the bioenergetic diagnostic output reached 100% across iterations for the same patient profile. Qualitative Evaluation Framework: Semantic precision and clinical relevance were audited using a 5 -item qualitative rubric (*Diagnostic Coherence, Mfn2/ICD-10 Correlation, Oxidative Stress Identification, Absence of Clinical Hallucination, and Translational Actionability*) independently scored by two domain experts (a histopathologist and a clinical endocrinologist) (see Figures 2, 3).

## 3 Results

### 3.1 Web interface architecture and local storage system (IndexedDB)

A decoupled, lightweight software prototype was successfully implemented, developed in HTML5, CSS3, and vanilla JavaScript, and designed to run entirely on the client side. The platform requires no external backend servers or data transmission to the cloud, implicitly ensuring compliance with privacy regulations and the protection of sensitive medical data (HIPAA/GDPR).

#### 3.1.1 Tab management and unique identification

The main interface (Supplementary Figure S1) was structured using a tabbed navigation system with independent active tabs, sequentially numbered from mitochondria_001 to 010 (representing the initial cohort of 10 clinical records, but this can be expanded to many more depending on usage scenarios). Each tab automatically assigns a unique storage key in IndexedDB. Multi-format support: The platform demonstrated the ability to instantly process and integrate high-resolution images in JPG, JPEG, PNG, and TIFF formats, corresponding to transmission electron microscopy (TEM), immunofluorescence, and immunohistochemistry photomicrographs. Persistence and Duplicate Control: When an image or record is loaded, the system verifies the unique key and displays the graphical confirmation indicator “LOADED DB.” The system includes a duplicate prevention algorithm that blocks accidental overwriting of the same patient key. Floating Action Buttons (FAB): Three persistent floating buttons were integrated that allow the immediate execution of commands: Image Upload, Local Transactional Save, and Clinical Record Display.

#### 3.1.2 Transactional integration of medical records

IndexedDB database, persistently retrieving and rendering the patient’s complete medical history. Integrated standardized fields include: Reason for consultation and current illness; Physical examination and vital signs (blood pressure, BMI, lipid profile); Complementary laboratory and imaging studies; and Diagnosis coded according to the ICD-10 international classification system.

#### 3.1.3 Photomicrographic management and labeling module

The image management module (Supplementary Figure S3) allows direct linking of photomicrographic files to the patient’s record. Each image displayed within the tab includes: Dedicated controls for saving, downloading, and inspecting the identification key in the Object Store. A rich text area for qualitative and quantitative tissue description (e.g., Mitofusin-2 labeling, nitrotyrosine levels, or degree of vacuolization on TEM).

### 3.2 Technical performance and evaluation of the local database

The computational performance of the prototype was evaluated by measuring latency times and browser memory usage efficiency during the manipulation of the cohort of 10 patients: Response Times and Latency: Reading and rendering a complete record (medical history in JSON + 4 micrographs in Base64 strings) from IndexedDB took an average of 35 milliseconds, demonstrating an ultra-fast transfer rate by not depending on network latencies or remote servers. Storage Stability: The object store stably handled more than 40 high-resolution micrographs without causing RAM saturation or drops in the browser’s main thread.

### 3.3 Prompts engineering framework for LLM

As an Artificial Intelligence component of the platform, the template of prompts structured in Chain of Thought was validated. The parameterized input of clinical variables (ICD-10, BMI) combined with molecular readings (Mfn2 expression, oxidative stress) allowed the Large Scale Language Models to generate coherent and structured interpretive reports in three main sections: Bioenergetic Diagnosis: Identification of the state of the mitochondrial network (fusion vs. fragmentation).

#### 3.3.1 Lipotoxic risk stratification

Qualitative scoring of oxidative damage based on reported nitrotyrosine levels to enable clinical correlation for the treating medical team regarding the impact of mitochondrial status on the patient’s cardiovascular and metabolic risk.

## 4 Discussion

The integration of Artificial Intelligence (AI) into contemporary clinical practice poses a fundamental dilemma in medical decision-making. On the one hand, complex Deep Learning (DL) architectures, such as convolutional neural networks (CNNs) or recurrent neural networks (RNNs), have demonstrated an unprecedented capacity to identify multivariate patterns in large volumes of data. However, as Bjerring et al. (2025) and Zednik (2021) point out, these models often function as structurally opaque “black boxes,” where high predictive accuracy is achieved at the cost of losing mechanistic interpretability and causal traceability.

In healthcare, algorithmic opacity introduces significant ethical and practical challenges. Healthcare professionals cannot base informed consent or risk stratification on recommendations whose pathophysiological basis remains inaccessible (Amann et al., 2020; Funer, 2022). As Coupland et al. (2025) demonstrated in longitudinal health data settings, although differential diagnosis models can outperform linear approaches in predicting outcomes, post-hoc explanations (such as SHAP scores) often fail to match the actual biological causal relationships.

Prompt engineering framework for LLMs was chosen, coupled with a transparent and decentralized web architecture. The Chain of Thought technique (Wei et al., 2022) used allows for the explicit definition of each step in the contextual reasoning of LLMs in medicine (Nori et al., 2023; Clusmann et al., 2023), ensuring the traceability of algorithmic inference and confidence in clinical decision-making (Amann et al., 2020). This allows the physician to audit, accept, modify, or reject the algorithmic suggestion, thus guaranteeing human-in-the-loop oversight and preserving the therapeutic alliance (Funer, 2022), and facilitating their work and practice with a secure tool that will free up time to dedicate to the patient and improve healthcare.

In the computational biomedical literature, there are valuable developments focused on the morphometric and ultrastructural characterization of mitochondria. Tools such as MitoMo (Zahedi et al., 2018) have enabled the automated classification of cell health and oxidative stress states in live cell cultures. Similarly, specialized software developed by Fischer et al. (2020), Khan et al. (2022), and Franco-Barranco et al. (2022) has facilitated the quantification of molecular alterations in *in vitro* experimental models. More recently, generative and deep learning models such as MiShape and MoDL (Suga et al., 2023; Punnakkal et al., 2023; Tsutsumi et al., 2023; Ding et al., 2024) have achieved three-dimensional reconstructions and accurate segmentation of the mitochondrial network from fluorescence and electron microscopy. However, the vast majority of these platforms share a common translational limitation: they are isolated from the patient’s clinical context. They operate in a way that focuses exclusively on the image or biological sample, omitting the medical history, vital signs, lipid profile, and diagnostic coding (ICD-10). Unlike these isolated models, the main strength of our software prototype lies not in training a computer vision segmentation network, but in its multimodal integration architecture, aligned with emerging trends in general-purpose medical AI (Moor et al., 2023). The HTML5/IndexedDB platform acts as a bridge between subcellular histopathological findings (Mfn2 expression, nitrotyrosine, and TEM ultrastructure) and the obese patient’s medical record. This provides clinicians with a unified and accessible query tool that contextualizes biological data within the individual’s overall pathological picture.

A crucial aspect discussed in modern medical informatics is the custody and privacy of health data. Centralizing medical records and high-resolution images on remote servers or cloud architectures exposes the system to cybersecurity risks and regulatory gaps regarding patient confidentiality. The development of our platform using the IndexedDB API demonstrates the feasibility of running a 100% client -side clinical and biomedical imaging management system. This technical approach, supported by the principles of Privacy by Design in locally processed web applications (Mascetti and Bettini, 2021) and evaluated in decentralized medical record architectures (Gasseller and Smith, 2022), offers three strategic advantages:

1. Privacy by design (privacy by design): medical records and photomicrographs remain in the user’s browser’s local memory, eliminating the risk of interception or leakage of sensitive data in transit to external servers.
2. Low latency and operational efficiency: with average response times of 35 ms for reading complete files (coinciding with the transactional efficiency and reduced latency analyses in local clients reported by Gasseller and Smith, 2022), the system allows smooth navigation between active tabs without requiring expensive server infrastructure.
3. The application can function in clinical environments with limited internet connectivity, democratizing its use in healthcare centers with limited resources.

Obesity represents an enormous burden on global health systems due to its chronic non-communicable complications (type 2 diabetes, cardiovascular disease, and cancer) (World Health Organization, 2017, 2024). Mitochondrial dysfunction in key metabolic tissues constitutes a central mechanistic link between lipotoxicity and the development of endocrine complications such as insulin resistance (Dudek, 2020) and placental developmental abnormalities (Fisher et al., 2021). The incorporation of digital tools and AI into clinical practice has the potential to transform the early stratification of this risk and the personalization of nutritional and pharmacological interventions (Azmi et al., 2025). However, as Clark et al. (2021), Funer (2022), and Coupland et al. (2025), the unrestricted deployment of technology in healthcare can amplify pre-existing inequities if the digital divide and the social determinants of health are not addressed. Medical AI tools risk benefiting only socioeconomic sectors with access to advanced technological infrastructure. In this regard, a prototype of lightweight, open-source software based on universal web standards (HTML5/IndexedDB) constitutes an equitable proposal. By not requiring supercomputers or specialized hardware for inferring complex models, it facilitates digital literacy and access to diagnostic support tools in marginalized or rural areas. The integration of conventional clinical data with mitochondrial biomarkers in an intuitive interface lays the groundwork for progressively updating maternal-fetal, pediatric, and adult healthcare guidelines, promoting a more inclusive and transparent translational precision medicine.

## Data Availability

The original contributions presented in the study are included in the article/Supplementary material, further inquiries can be directed to the corresponding author.

## Footnotes

1
https://github.com/karenth-rodriguez/mitochondrial-llm-platform
