# Supplementary material for: System architecture and interactive LLM prompt framework for the integration of clinical and mitochondrial data in obesity: a software prototype and feasibility study
Source: Front Artif Intell. 2026 Sep 10;9:1923426. doi: 10.3389/frai.2026.1923426 (PMC13601289; doi:10.3389/frai.2026.1923426)
Supplement: Supplementary file 1 [file Supplementary_file_1.pdf]

Supplementary Material S1

The complete, fully functional source code for the client-side web application (developed in vanilla HTML5, CSS3, and JavaScript) as well as the underlying IndexedDB database schemas utilized in this study are available to support reproducibility and further open-source development. To streamline the main manuscript and prioritize scientific contributions, all source code blocks have been transferred to this supplementary file and deposited in a public GitHub repository: https://github.com/karenth-rodriguez/mitochondrial-llm-platform file:///C:/Users/karen/OneDrive/Escritorio/INTELIGENCIA%20ARTIFICIAL/ia_F57N.html

Repository Contents:

1. `index.html`: User interface layout for clinical record data entry and microphotograph display.
2. `app.js`: Client-side logic for IndexedDB local storage transactions, anonymized patient management, and LLM prompt integration.
3. `schema.json`: Structural definition for offline patient clinical and mitochondrial data schemas.

IMAGES VISIBLE IMMEDIATELY

🔬 **4 ACTIVE tabs**

🔢 **mitochondria_001 → 010 PER TAB**

🖼️ **JPG, JPEG, TIFF**

🔑 **UNIQUE CODE KEY**

✅ **Badge "LOADED DB"**

🗄️ **INDEFINITE SAVE ON**

🩺 **PERSISTENT MEDICAL HISTORIES**

EXCLUSION OF DUPLICATES

3 FLOATING SAVE BUTTONS

🧬 MITOCHONDRIA ALGORITHM - **INDEXEDDB 100% ENABLED**

👁️ IMAGES VISIBLE IMMEDIATELY

💾 FLOATING BUTTONS ENABLED

HTML SOURCE CODE

```
<!DOCTYPE html>
<html lang="es">
<head>
<meta charset="UTF-8">
<meta name="viewport" content="width=device-width, initial-scale=1.0">
<title> 🧬 MITOCHONDRIA ALGORITHM - IMAGES + MEDICAL HISTORY **ACTIVATED**</title>
<style>
* { margin: 0; padding: 0; box-sizing: border-box; }
body {
font-family: 'Segoe UI', Tahoma, Geneva, Verdana, sans-serif;
background: linear-gradient(135deg, #0f0f23 0%, #1a1a2e 50%, #16213e 100%);
min-height: 100vh; padding: 20px; color: #333;
}
.container {
max-width: 1900px; margin: 0 self;
background: rgba(255,255,255,0.95);
border-radius: 40px; padding: 60px;
box-shadow: 0 50px 180px rgba(0,0,0,0.5);
}
h1 {
text-align: center;
background: linear-gradient(135deg, #ff6b6b, #4ecdc4, #45b7d1, #28a745, #ffd93d);
-webkit-background-clip: text; -webkit-text-fill-color: transparent;
font-size: 60px; margin-bottom: 40px; animation: glow 2s infinite alternate;
}
@keyframes glow { from { text-shadow: 0 0 30px #fff; } to { text-shadow: 0 0 30px #28a745; } }
```

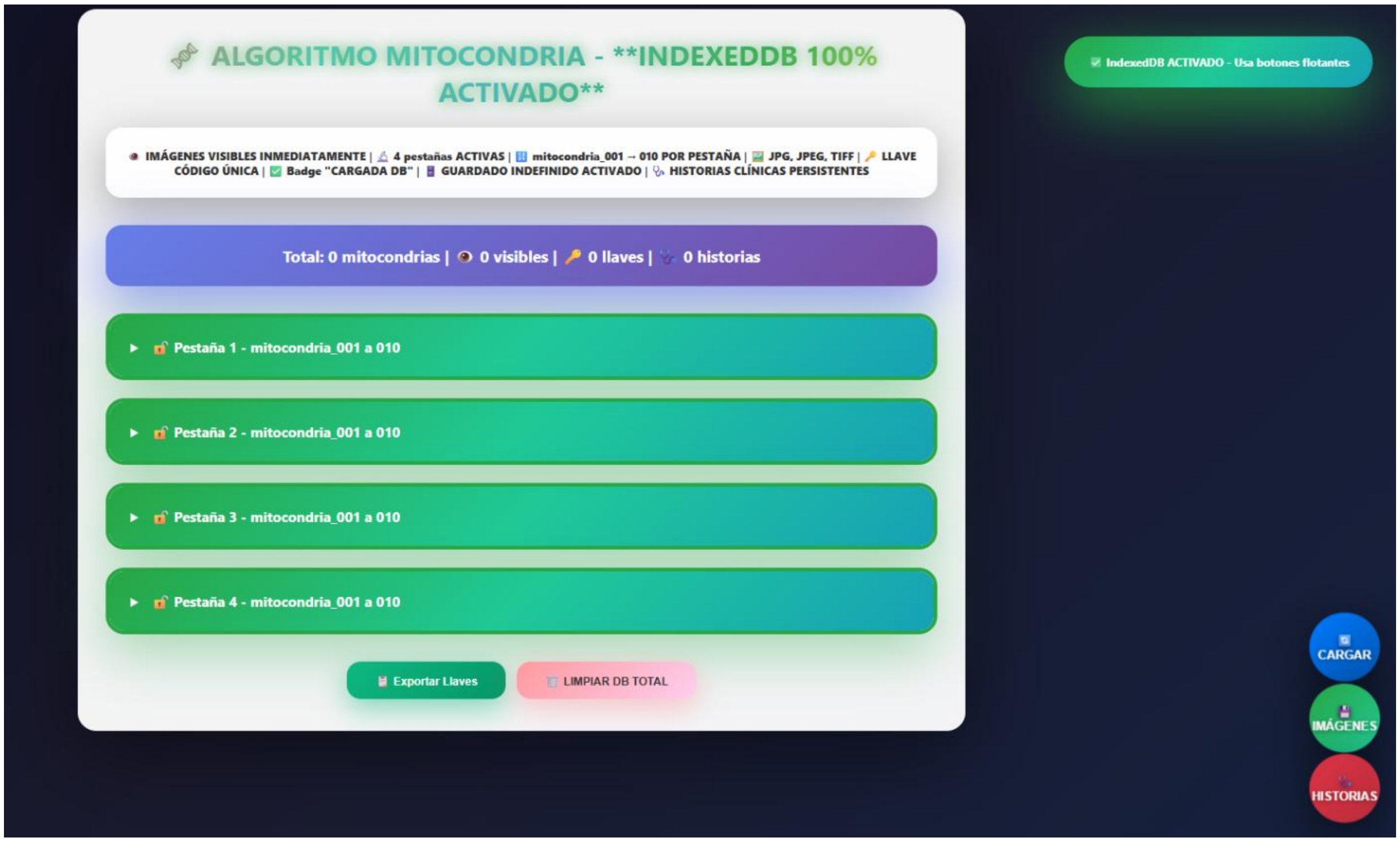

Figure 4: *HTML algorithm that displays immediately visible images, with 4 active tabs, each tab numbered from 001 to 010 for 10 patients, 3 floating save buttons (load images, save images, view medical records), and each tab and image has a key with a unique save code to add images and then easily identify them using the key in the search engin*

ALGORITHM OF FIGURE 4:

```
/* 💾 BUTTONS ACTIVATED - FLOATING */
.control-buttons {
position: fixed; bottom: 70px; right: 70px; display: flex; flex-direction: column;
gap: 15px; z-index: 15000;
}
.btn-control {
width: 140px; height: 140px; border-radius: 50%; border:none;
font-size: 24px; font-weight: bold; cursor: pointer; colour: white;
box-shadow: 0 30px 100px rgba(0,0,0,0.6); transition: all 0.4s;
display: flex; flex-direction: column; align-items: center; justify-content: center;
text-shadow: 0 2px 10px rgba(0,0,0,0.5);
```

```css
}
.btn-save-img { background: linear-gradient(135deg, #28a745, #20c997); animation: pulse-green 2s infinite; }
.btn-save-hc { background: linear-gradient(135deg, #dc3545, #bb2d3b); animation: pulse-red 2s infinite; }
.btn-load-all { background: linear-gradient(135deg, #007bff, #0056b3); animation: pulse-blue 2s infinite; }
.btn-control:hover { transform: scale(1.2) translateY(-15px) !important; box-shadow: 0 50px 150px rgba(0,0,0,0.8); }
@keyframes pulse-green { 0%,100%{transform:scale(1);} 50%{transform:scale(1.08);} }
@keyframes pulse-red { 0%,100%{transform:scale(1);} 50%{transform:scale(1.08);} }
@keyframes pulse-blue { 0%,100%{transform:scale(1);} 50%{transform:scale(1.08);} }

.status {
position: fixed; top: 80px; right: 80px; background: rgba(40,167,69,0.98);
colour: white; padding: 40px 50px; border-radius: 55px; font-weight: bold; font-size: 24px;
box-shadow: 0 40px 130px rgba(40,167,69,0.85); z-index: 9999; min-width: 380px;
text-align: center;
}
.status.error { background: linear-gradient(135deg, #dc3545, #c82333); }
.status.unlocked { background: linear-gradient(135deg, #28a745, #20c997, #17a2b8) !important; animation: press 1s infinite; }

.info { text-align: center; font-size: 24px; margin-bottom: 60px; font-weight: 600;
background: rgba(255,255,255,0.9); padding: 45px; border-radius: 35px;
box-shadow: 0 30px 100px rgba(0,0,0,0.3);
}
/* 🩺 MEDICAL HISTORY STYLES */
.btn-history {
background: linear-gradient(135deg, #dc3545, #bb2d3b, #8b0000);
colour: white; padding: 20px 40px; border:none; border-radius: 25px;
font-size: 22px; font-weight: bold; cursor: pointer; margin: 20px 0;
box-shadow: 0 20px 60px rgba(220,53,69,0.6); transition: all 0.4s;
}
.btn-historia:hover {
transform: translateY(-10px) scale(1.05); box-shadow: 0 30px 90px rgba(220,53,69,0.9);
}
.modal-history {
display: none; position: fixed; z-index: 30000; left: 0; top: 0;
width: 100%; height: 100%; background: rgba(0,0,0,0.9); backdrop-filter: blur(10px);
}
.modal-content {
background: linear-gradient(145deg, #ffffff, #f8f9fa); margin: 5% auto; padding: 40px;
```

```
border-radius: 35px; width: 90%; max-width: 1200px; max-height: 90vh; overflow-y: self;
box-shadow: 0 50px 180px rgba(0,0,0,0.8); border: 6px solid #dc3545;
}
.close-modal { color: #dc3545; float: right; font-size: 40px; font-weight: bold; cursor: pointer; }
.form-group { margin-bottom: 25px; }
.form-group label { display: block; font-weight: bold; color: #333; margin-bottom: 10px; font-size: 18px; }
.form-group input, .form-group textarea, .form-group select {
width: 100%; padding: 18px; border: 3px solid #dc3545; border-radius: 20px;
font-size: 16px; transition: all 0.3s; background: rgba(220,53,69,0.05);
}
.form-group input:focus, .form-group textarea:focus, .form-group select:focus {
outline: none; border-color: #bb2d3b; box-shadow: 0 0 20px rgba(220,53,69,0.3);
transform: scale(1.02);
}
.vital-signs { display: grid; grid-template-columns: repeat(auto-fit, minmax(200px, 1fr)); gap: 20px; }
.btn-guardar-historia {
background: linear-gradient(135deg, #28a745, #20c997); colour: white; padding: 20px 50px;
border:none; border-radius: 25px; font-size: 20px; font-weight: bold; cursor: pointer;
box-shadow: 0 20px 60px rgba(40,167,69,0.6); width: 100%; margin-top: 20px;
}
.btn-save-history:hover { transform: translateY(-5px); box-shadow: 0 25px 80px rgba(40,167,69,0.8); }
.saved-history {
background: linear-gradient(135deg, #28a745, #20c997); colour: white; padding: 15px;
border-radius: 20px; margin-top: 15px; font-weight: bold; display: none;
}
.cie10-select { height: 120px; }

details { border: 8px solid #28a745; margin: 40px 0; border-radius: 40px;
background: linear-gradient(145deg, #f8fff9, #e8f5e8); overflow: hidden;
transition: all 0.6s; box-shadow: 0 35px 120px rgba(40,167,69,0.4);
}
details[open] { transform: translateY(-25px); box-shadow: 0 65px 220px rgba(40,167,69,0.6); }
summary { cursor: pointer; font-weight: bold; padding: 45px;
background: linear-gradient(135deg, #28a745, #20c997, #17a2b8); colour: white;
font-size: 30px; transition: all 0.5s;
}
summary::-webkit-details-marker { display: none; }
.tab-content { padding: 50px; }
```

```css
.images-container {
display: grid;
grid-template-columns: repeat(auto-fill, minmax(420px, 1fr));
gap: 35px; margin-top: 35px; min-height: 500px;
}
.image-item {
text-align: center;
background: linear-gradient(145deg, #ffffff, #f8f9fa);
padding: 35px;
border-radius: 30px;
box-shadow: 0 25px 90px rgba(0,0,0,0.25);
transition: all 0.6s ease;
position: relative;
border: 4px solid transparent;
overflow: hidden;
}
.image-item:hover {
transform: translateY(-25px) scale(1.08);
box-shadow: 0 55px 180px rgba(0,0,0,0.45);
border-color: #3b82f6;
}
.image-item.retrieved::before {
content: " ✅ LOADED DB";
position: absolute;
top: 15px;
right: 15px;
background: linear-gradient(135deg, #10b981, #059669);
color: white;
padding: 8px 18px;
border-radius: 20px;
font-size: 14px;
font-weight: bold;
box-shadow: 0 8px 25px rgba(16,185,129,0.6);
z-index: 10;
}
.image-preview {
max-width: 100%;
```

```
max-height: 350px;
border-radius: 25px;
border: 6px solid #10b981;
box-shadow: 0 25px 90px rgba(16,185,129,0.6);
transition: all 0.4s ease;
}
.imagen-item:hover .imagen-preview {
transform: scale(1.05);
box-shadow: 0 35px 120px rgba(16,185,129,0.8);
}
.image-label {
font-weight: 900;
color: #10b981;
margin: 25px 0;
font-size: 28px;
background: linear-gradient(135deg, #d1fae5, #a7f3d0, #10b981);
-webkit-background-clip: text;
-webkit-text-fill-color: transparent;
padding: 20px;
border-radius: 20px;
}
.key-code {
background: linear-gradient(135deg, #1e3a8a, #3b82f6, #8b5cf6);
color: white;
font-family: 'Courier New', monospace;
font-weight: bold;
font-size: 16px;
padding: 12px 20px;
border-radius: 20px;
margin: 15px 0;
letter-spacing: 1.5px;
box-shadow: 0 12px 45px rgba(59,130,246,0.6);
word-break: break-all;
}
.description {
margin: 15px 0;
font-size: 14px;
color: #555;
```

```
text-align: left;
padding: 12px;
background: linear-gradient(145deg, #f8f9fa, #e9ecef);
border-radius: 15px;
border-left: 4px solid #10b981;
}
.btn-group-item {
display: grid;
grid-template-columns: repeat(4, 1fr);
gap: 15px;
margin-top: 20px;
}
.btn-item {
padding: 18px 25px;
border: none;
border-radius: 20px;
cursor: pointer;
font-weight: bold;
font-size: 15px;
transition: all 0.4s;
box-shadow: 0 12px 40px rgba(0,0,0,0.25);
}
.btn-download { background: linear-gradient(135deg, #007bff, #0056b3); colour: white; }
.btn-copy { background: linear-gradient(135deg, #8b5cf6, #a78bfa); colour: white; }
.btn-save { background: linear-gradient(135deg, #10b981, #059669); colour: white; }
.btn-delete { background: linear-gradient(135deg, #dc3545, #c82333); colour: white; }
.btn-item:hover { transform: translateY(-8px) scale(1.08); box-shadow: 0 20px 60px rgba(0,0,0,0.4); }

input[type="file"], input[type="text"], textarea {
margin: 20px 0;
padding: 25px;
border: 5px dashed #10b981;
border-radius: 25px;
background: rgba(16,185,129,0.1);
width: 100%;
font-size: 18px;
transition: all 0.5s;
cursor: pointer;
```

```
font-family: inherit;
}
input[type="file"]:hover, input[type="text"]:hover, textarea:hover {
background: rgba(16,185,129,0.25);
transform: scale(1.02);
box-shadow: 0 20px 70px rgba(16,185,129,0.5);
}
.counter {
font-weight: bold;
color: #10b981;
margin-bottom: 30px;
font-size: 30px;
padding: 35px;
background: linear-gradient(135deg, #d1fae5, #a7f3d0);
border-radius: 30px;
text-align: center;
box-shadow: 0 25px 90px rgba(16,185,129,0.6);
}
.total-stats {
text-align: center;
margin: 60px 0;
padding: 45px;
background: linear-gradient(135deg, #667eea, #764ba2);
color: white;
border-radius: 35px;
font-size: 34px;
font-weight: bold;
box-shadow: 0 35px 120px rgba(102,126,234,0.8);
}

.repetitive-alert {
position: fixed; top: 50%; left: 50%; transform: translate(-50%, -50%);
background: linear-gradient(135deg, #ff6b6b, #ff8e8e);
color: white;
padding: 50px 70px;
border-radius: 35px;
font-size: 24px;
font-weight: bold;
```

```
text-align: center;
box-shadow: 0 50px 180px rgba(255,107,107,0.9);
z-index: 20000;
border: 6px solid rgba(255,255,255,0.3);
animation: shake 0.6s infinite;
}
@keyframes shake {
0%,100%{transform: translate(-50%, -50%) translateX(0);}
25%{transform: translate(-50%, -50%) translateX(-8px);}
75%{transform: translate(-50%, -50%) translateX(8px);}
}
</style>
</head>
<body>
<!-- 💾 FLOATING BUTTONS ENABLED -->
<div class="control-buttons">
<button class="btn-control btn-load-all" title=" 🔄 LOAD ALL from IndexedDB" onclick="loadAllFromDB()">
    🔄 <br><span>LOAD</span>
</button>
<button class="btn-control btn-save-img" title=" 💾 SAVE Images" onclick="guardarImagenesDB()">
    💾 <br><span>IMAGES</span>
</button>
<button class="btn-control btn-save-hc" title=" 🩺 SAVE Medical Records" onclick="guardarHistoriasDB()">
    🩺 <br><span>STORIES</span>
</button>
</div>

<div class="status unlocked" id="status"> 🔄 IndexedDB ENABLED - Ready to save/load...</div>

<div class="container">
<h1> 🧬 MITOCHONDRIA ALGORITHM - **INDEXEDDB 100% ENABLED**</h1>
<p class="info">
    👁️ <strong>IMAGES VISIBLE IMMEDIATELY</strong> |
    🔬 <strong>4 ACTIVE tabs</strong> |
    🔢 <strong>mitochondria_001 → 010 PER TAB</strong> |
    🖼️ <strong>JPG, JPEG, TIFF</strong> |
    🔑 <strong>UNIQUE CODE KEY</strong> |
```

```
✅ <strong>Badge "LOADED DB"</strong> |

🗄️ <strong>INDEFINITE SAVED ON</strong> |

🩺 <strong>PERSISTENT MEDICAL HISTORIES</strong>

</p>

<div class="total-stats" id="totalStats">Total: 0 mitochondria | 👁️ 0 visible | 🔑 0 keys | 🩺 0 stories</div>

<!-- TABS 1-4 -->
<details id="tab1" open><summary> 🔓 Tab 1 - mitochondria_001 to 010</summary>
<div class="tab-content">
<div class="counter">Visible: <span id="count1">0</span>/10</div>
<button class="btn-historia" onclick="abrirHistoriaClinica(1)"> 🩺 NEW MEDICAL HISTORY</button>
<div id="history1" class="saved-history"></div>
<input type="text" id="nombrePestaña1" placeholder="Tab 1 Name">
<textarea id="descPestana1" rows="2" placeholder="Description for images in Tab 1..."></textarea>
<input type="file" id="file1" accept="image/jpeg,image/jpg,image/tiff,image/tif" multiple onchange="loadImages(1, this.files)">
<div id="gallery1" class="images-container"></div>
</div>
</details>

<details id="tab2" open><summary> 🔓 Tab 2 - mitochondria_001 to 010</summary>
<div class="tab-content">
<div class="counter">Visible: <span id="count2">0</span>/10</div>
<button class="btn-historia" onclick="abrirHistoriaClinica(2)"> 🩺 NEW MEDICAL HISTORY</button>
<div id="history2" class="saved-history"></div>
<input type="text" id="nombrePestaña2" placeholder="Nombre de tab 2">
<textarea id="descPestana2" rows="2" placeholder="Description for images in Tab 2..."></textarea>
<input type="file" id="file2" accept="image/jpeg,image/jpg,image/tiff,image/tif" multiple onchange="loadImages(2, this.files)">
<div id="gallery2" class="images-container"></div>
</div>
</details>

<details id="tab3" open><summary> 🔓 Tab 3 - mitochondria_001 to 010</summary>
<div class="tab-content">
<div class="counter">Visible: <span id="count3">0</span>/10</div>
<button class="btn-historia" onclick="abrirHistoriaClinica(3)"> 🩺 NEW MEDICAL HISTORY</button>
<div id="history3" class="saved-history"></div>
```

```html
<input type="text" id="nombrePestaña3" placeholder="Nombre de tab 3">

<textarea id="descPestana3" rows="2" placeholder="Description for images of Tab 3..."></textarea>

<input type="file" id="file3" accept="image/jpeg,image/jpg,image/tiff,image/tif" multiple onchange="loadImages(3, this.files)">

<div id="gallery3" class="images-container"></div>

</div>

</details>


<details id="tab4" open><summary> 🔓 Tab 4 - mitochondria_001 to 010</summary>

<div class="tab-content">

<div class="counter">Visible: <span id="count4">0</span>/10</div>

<button class="btn-historia" onclick="abrirHistoriaClinica(4)"> 🩺 NEW MEDICAL HISTORY</button>

<div id="history4" class="saved-history"></div>

<input type="text" id="nombrePestaña4" placeholder="Tab 4 Name">

<textarea id="descPestana4" rows="2" placeholder="Description for images of Tab 4..."></textarea>

<input type="file" id="file4" accept="image/jpeg,image/jpg,image/tiff,image/tif" multiple onchange="loadImages(4, this.files)">

<div id="gallery4" class="images-container"></div>

</div>

</details>


<div style="text-align: center; margin-top: 50px;">

<button onclick="exportKeys()" class="btn-item" style="background: linear-gradient(135deg, #10b981, #059669); color: white; padding: 25px 60px; border-radius: 30px; font-size: 24px; margin: 10px; box-shadow: 0 20px 60px rgba(16,185,129,0.5);"> 📋 Export Keys</button>

<button onclick="clearIndexedDB()" class="btn-item" style="background: linear-gradient(135deg, #ff9a9e, #fecfef); color: #333; padding: 25px 60px; border-radius: 30px; font-size: 24px; margin: 10px; box-shadow: 0 20px 60px rgba(255,154,158,0.5);"> 🗑️ CLEAR DB TOTAL</button>

</div>

</div>


<!-- 🩺 MODAL MEDICAL HISTORY -->

<div id="modalHistory" class="modal-history">

<div class="modal-content">

<span class="close-modal" onclick="closeClinicalHistory()">&times;</span>

<h2 style="color: #dc3545; text-align: center; margin-bottom: 30px; font-size: 32px;"> 🩺 CLINICAL HISTORY</h2>


<div class="form-group">

<label>Tab:</label>

<input type="text" id="History tab" readonly>

</div>
```

```
<div class="form-group">
<label>Patient Name:</label>
<input type="text" id="PatientName">
</div>

<div style="display: grid; grid-template-columns: 1fr 1fr; gap: 20px;">
<div class="form-group">
<label>ID Card:</label>
<input type="text" id="Patient ID">
</div>
<div class="form-group">
<label>Age:</label>
<input type="number" id="PatientAge" min="0" max="120">
</div>
</div>

<div class="form-group">
<label>Date:</label>
<input type="date" id="querydate">
</div>

<div class="form-group">
<label>Reason for Consultation:</label>
<textarea id="queryreason" rows="3"></textarea>
</div>

<div class="form-group">
<label>Current Illness:</label>
<textarea id="currentdisease" rows="4"></textarea>
</div>

<div class="form-group">
<label>Physical Examination - Vital Signs:</label>
<div class="vital-signs">
<input type="text" id="tensionArterial" placeholder="Blood tension (ex: 120/80)">
<input type="text" id="Heart Rate" placeholder="HR (ex: 72 bpm)">
<input type="text" id="Respiratory Rate" placeholder="FR (ex: 16 rpm)">
```

```html
<input type="text" id="Oxygen saturation" placeholder="Sat O2 (ex: 98%)">
<input type="text" id="temperature" placeholder="Temperature (ex: 36.5°C)">
<input type="text" id="imc" placeholder="BMI (ex: 24.5)">
<input type="text" id="weight" placeholder="Weight (ex: 70 kg)">
<input type="text" id="size" placeholder="Size (ex: 1.70 m)">
</div>
</div>

<div class="form-group">
<label>Complementary Diagnostic Studies:</label>
<textarea id="estudiosComplementarios" rows="4"></textarea>
</div>

<div class="form-group">
<label>ICD-10 diagnoses:</label>
<select id="diagnosticosCIE10" class="cie10-select" multiple>
<option value="A00">A00 - Anger</option>
<option value="J45">J45 - Asthma</option>
<option value="E11">E11 - Type 2 Diabetes</option>
<option value="I10">I10 - Essential Hypertension</option>
<option value="K21">K21 - Gastroesophageal reflux</option>
<option value="M54">M54 - Dorsalgia</option>
<option value="N39">N39 - Urination disorders</option>
<option value="R10">R10 - Abdominal pain</option>
<option value="Z00">Z00 - Health exams</option>
</select>
</div>

<button class="btn-guardar-historia" onclick="guardarHistoriaClinica()">💾 SAVE MEDICAL HISTORY</button>
</div>
</div>
```

<script>

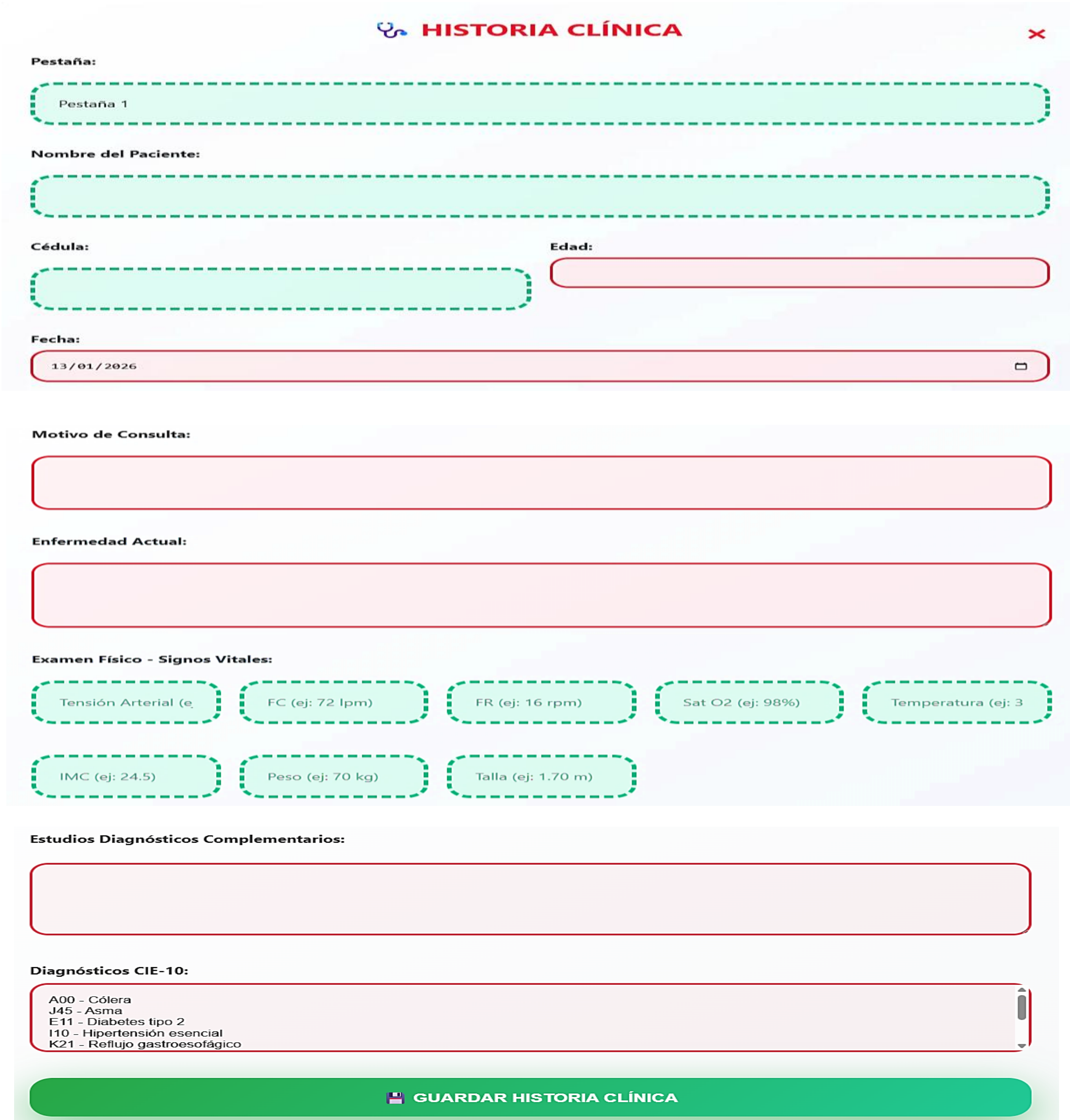

Figure 5*: HTML algorithm that displays images of the complete medical record when each tab is opened, with patient data, medical record data (reason for consultation, current illness, physical examination, vital signs, complementary studies and diagnoses)*

ALGORITHM OF FIGURE 5:

```
// 🌐 GLOBAL VARIABLES
let db, tabcounters = Array(4).fill(0), allImages = [],
imageUrls = new Map(), llavesCodigos = new Set(), dbIniciada = false;
let clinicalhistories = {};

// 🔑 GENERATE UNIQUE KEY
function generateKeyCode(tab, number) {
const timestamp = Date.now().toString(36).toUpperCase().slice(-6);
const random = Math.random().toString(36).toUpperCase().slice(-6);
return `KEY-${tab.toString().padStart(2,'0')}-${number.toString().padStart(3,'0')}-${timestamp}-${random}`;
}

// 🗄 INITIALIZE INDEXEDDB WITH BUTTONS ENABLED
function initIndexedDB() {
const request = indexedDB.open('MitochondriaDB_Pro', 10);

request.onsuccess = (e) => {
db = e.target.result;
dbIniciada = true;
document.getElementById('status').innerHTML = '✅ IndexedDB ENABLED - Use floating buttons';
document.getElementById('status').className = 'status unlocked';
};

request.onupgradeneeded = (e) => {
db = e.target.result;

// Images
if (!db.objectStoreNames.contains('mitochondria')) {
const storeImg = db.createObjectStore('mitochondria', { keyPath: 'id' });
storeImg.createIndex('tab', 'tab');
storeImg.createIndex('keycode', 'keycode');
}

// Medical records
if (!db.objectStoreNames.contains('ClinicalHistories')) {
const storeHC = db.createObjectStore('clinicalhistories', { keyPath: 'id' });
```

```
storeHC.createIndex('tab', 'tab');
}
};

request.onerror = () => {
document.getElementById('status').innerHTML = '❌ IndexedDB Error';
document.getElementById('status').className = 'status error';
};
}

// 💾 BUTTON 1: SAVE IMAGES TO DB
async function saveImagesDB() {
if (!dbStarted || !db || allImages.length === 0) {
showAlert('❌ No images to save');
return;
}

let guardadas = 0;
const transaction = db.transaction(['mitochondria'], 'readwrite');
const store = transaction.objectStore('mitochondria');

for (let imgData of allImages) {
try {
imgData.descripcion = document.getElementById(`descPestana${imgData.pestana}`)?.value || imgData.descripcion || '';
await store.put(imgData);
keysCodes.add(imgData.keyCode);
saved++;
} catch(e) {
console.error('Error saving image:', e);
}
}

document.getElementById('status').innerHTML = `✅ Saved ${saved} IMAGES in IndexedDB 💾 `;
showAlert(`✅ ${saved} IMAGES saved INDEFINITELY`);
}

// 🩺 BUTTON 2: SAVE STORIES IN DB
```

```
async function saveDBHistories() {
if (!dbStarted || !db || Object.keys(ClinicalHistories).length === 0) {
showAlert(' ❌ No medical records to save');
return;
}

const transaction = db.transaction(['ClinicalHistories'], 'readwrite');
const store = transaction.objectStore('ClinicalHistories');
let guardadas = 0;

for (let pestana in historiasClinicas) {
try {
const historia = {...historiasClinicas[pestana], id: `historia_${pestana}`};
await store.put(history);
saved++;
} catch(e) {
console.error('Error saving history:', e);
}
}

document.getElementById('status').innerHTML = ` ✅ Saved ${saved} STORIES in IndexedDB 🩺 `;
showAlert(` ✅ ${saved} STORIES saved INDEFINITELY`);
}
```

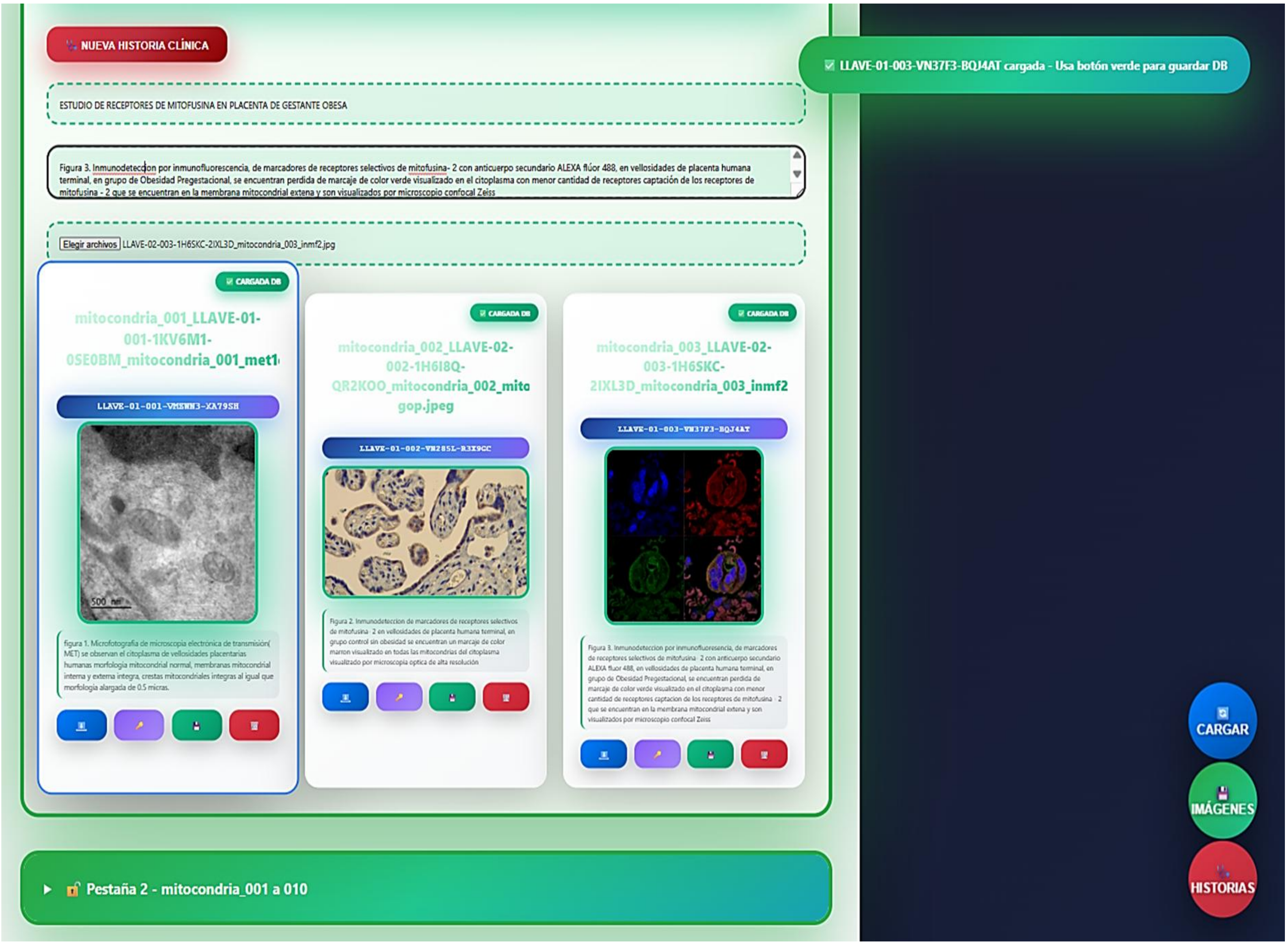

Figure 6: *HTML algorithm that displays images from the medical record in each tab, where histopathology images, laboratory results, etc., can be attached. Each image has buttons (save, download, save and download key) as well as a field to describe each added image.*

ALGORITHM FOR FIGURE 6:

```
// 🔄 BUTTON 3: LOAD EVERYTHING FROM DB
async function loadAllFromDB() {
if (!dbIniciada || !db) {
showAlert(' ❌ IndexedDB not available');
return;
}
```

```
// Upload images
const txImg = db.transaction(['mitochondria'], 'readonly');
const storeImg = txImg.objectStore('mitochondria');
storeImg.getAll().onsuccess = (e) => {
allImages = e.target.result || [];
allImages.forEach(img => {
keysCodes.add(img.keyCode);
imageUrls.set(img.id, img.data);
const idx = img.pestana - 1;
if (idx >= 0 && idx < 4) {
countersPestana[idx] = Math.max(countersPestana[idx], img.number || 0);
}
});
showLoadedImages();
};

// Load stories
const txHC = db.transaction(['clinicalhistories'], 'readonly');
const storeHC = txHC.objectStore('clinicalhistories');
storeHC.getAll().onsuccess = (e) => {
const stories = e.target.result || [];
stories.forEach(story => {
clinicalhistories[history.tab] = history;
const div = document.getElementById(`historial${historia.pestana}`);
if (div && historia.nombrePaciente) {
div.innerHTML = `✅ ${historia.nombrePaciente} | ${new Date(historia.fechaGuardado).toLocaleString('es-ES')}`;
div.style.display = 'block';
}
});
updateStatistics();
document.getElementById('status').innerHTML = `✅ LOADED ALL: ${allImages.length} imgs + ${stories.length} stories`;
showAlert(`✅ ALL LOADED from IndexedDB`);
};
}

// 🩺 MEDICAL HISTORY FUNCTIONS
```

```
function openClinicHistory(tab) {
document.getElementById('pestanaHistoria').value = `Tab ${pestana}`;
document.getElementById('modalHistory').style.display = 'block';
document.body.style.overflow = 'hidden';

if (historiasClinicas[pestana]) {
const historia = historiasClinicas[pestana];
Object.keys(history).forEach(key => {
const el = document.getElementById(key);
if (el && key !== 'tap' && key !== 'id' && key !== 'saveDate') {
if (key === 'CIE10diagnostics') {
Array.from(el.options).forEach(opt => opt.selected = history[key].includes(opt.value));
} else {
el.value = historia[key] || '';
}
}
});
}
}

function closeMedicalHistory() {
document.getElementById('modalHistory').style.display = 'none';
document.body.style.overflow = 'auto';
}

function saveClinicalHistory() {
const tab = parseInt(document.getElementById('HistoryTab').value.match(/\d+/)[0]);
const history = {
eyelash,
patientName: document.getElementById('patientName').value,
cedulaPaciente: document.getElementById('cedulaPaciente').value,
PatientAge: document.getElementById('PatientAge').value,
querydate: document.getElementById('consultationdate').value,
queryreason: document.getElementById('queryreason').value,
currentillness: document.getElementById('currentillness').value,
tensionArterial: document.getElementById('tensionArterial').value,
heartrate: document.getElementById('heartrate').value,
respiratoryfrequency: document.getElementById('respiratoryfrequency').value,
```

```
saturacionOxigeno: document.getElementById('saturacionOxigeno').value,
temperature: document.getElementById('temperature').value,
imc: document.getElementById('imc').value,
weight: document.getElementById('weight').value,
size: document.getElementById('size').value,
ComplementaryStudies: document.getElementById('ComplementaryStudies').value,
diagnosticosCIE10: Array.from(document.getElementById('diagnosticosCIE10').selectedOptions).map(opt => opt.value),
SavedDate: new Date().toISOString()
};

clinicalhistories[tab] = history;
const div = document.getElementById(`history${tab}`);
div.innerHTML = `✅ ${historia.nombrePaciente || 'Paciente'} | ${new Date().toLocaleString('es-ES')}`;
div.style.display = 'block';

updateStatistics();
closeMedicalHistory();
document.getElementById('status').innerHTML = `🩺 History saved in memory - Use red button for DB`;
}

// 👁 IMAGE FUNCTIONS (NO CHANGES)
function showLoadedImages() {
for(let i = 1; i <= 4; i++) {
document.getElementById(`gallery${i}`).innerHTML = '';
document.getElementById(`count${i}`).textContent = '0';
}

tabcounters = Array(4).fill(0);
allImages.forEach(imgData => {
if (imgData.tab && imgData.data) {
const idx = imgData.pestana - 1;
pestanacounters[idx] = Math.max(pestanacounters[idx], imgData.number || 0);
if (!imageUrls.has(imgData.id)) imageUrls.set(imgData.id, imgData.data);

const url = imageUrls.get(imgData.id);
const gallery = document.getElementById(`gallery${imgData.pestana}`);
if (gallery) {
const element = createImageElement({...imgData, data: url});
```

```
element.classList.add('recovered');
gallery.appendChild(item);
}
}
});

for(let i = 0; i < 4; i++) {
document.getElementById(`count${i+1}`).textContent = countersPestana[i];
}
updateStatistics();
}

function createImageElement(imgData) {
const item = document.createElement('div');
item.className = 'item-image';
item.dataset.imgId = imgData.id;
item.innerHTML = `
<div class="imagen-label">${imgData.nombreCompleto}</div>
<div class="llave-codigo">${imgData.codigoLlave || 'Sin llave'}</div>
<img src="${imgData.data}" class="preview-image" alt="${imgData.fullName}" loading="lazy">
<div class="descripcion">${imgData.descripcion || 'No description'}</div>
<div class="btn-group-item">
<a href="${imgData.data}" download="${imgData.keycode}_${imgData.fullname}" class="btn-item btn-download"> ⬇️ </a>
<button class="btn-item btn-copy" onclick="copyKey('${imgData.id}')"> 🔑 </button>
<button class="btn-item btn-save" onclick="downloadIndividual('${imgData.id}')"> 💾 </button>
<button class="btn-item btn-delete" onclick="deleteImage('${imgData.id}', ${imgData.pestana || 1})"> 🗑️ </button>
</div>
`;
return item;
}

async function loadImages(tab, files) {
const tabIdx = tab - 1;
const available = 10 - tabcounters[tapIdx];

if (available <= 0) {
showAlert(` ❌ Tab ${tab} FULL`);
```

```
return;
}
Array.from(files).slice(0, available).forEach(async (file) => {
const number = ++countersPestana[PestanaIdx];
const id = `myth_${tab}_${number}_${Date.now()}_${Math.random().toString(36).slice(2)}`;
const keycode = generateKeyCode(tab, number);

const reader = new FileReader();
reader.onload = async(e) => {
const imgData = {
id, name: file.name, fullName: `mitochondria_${numero.toString().padStart(3,'0')}_${file.name}`,
keycode, data: e.target.result, tab, number, date: new Date().toISOString(),
description: document.getElementById(`descPestana${pestana}`)?.value || ''
};

allImages.push(imgData);
keysCodes.add(keyCode);
imageUrls.set(id, e.target.result);

const gallery = document.getElementById(`gallery${tab}`);
const element = createImageElement(imgData);
element.classList.add('recovered');
gallery.appendChild(item);

document.getElementById(`count${tab}`).textContent = number;
updateStatistics();
document.getElementById('status').innerHTML = `✅ ${keycode} loaded - Use green button to save DB`;
};
reader.readAsDataURL(file);
});
}

// UTILITY FUNCTIONS
function updateStats() {
document.getElementById('totalStats').innerHTML =
`Total: <strong>${todasLasImagenes.length}</strong> mitochondrias | 👁 <strong>${todasLasImagenes.length}</strong> visibles | 🔑 <strong>${llavesCodigos.size}</strong> keys | 🩺 <strong>${Object.keys(historiasClinicas).length}</strong> stories`;
}
```

```
function copyKey(id) {
const img = allImages.find(i => i.id === id);
if (img?.codigoLlave) {
navigator.clipboard.writeText(img.keycode).then(() => {
document.getElementById('status').innerHTML = ` 🔑 Copied: ${img.keycode.slice(0,20)}...`;
setTimeout(updateStats, 1500);
});
}
}
function deleteImage(id, tab) {
if (confirm('Delete image?')) {
const imgData = allImages.find(i => i.id === id);
if (imgData) llavesCodigos.delete(imgData.codigoLlave);
document.querySelector(`[data-img-id="${id}"]`).remove();
const idx = allImages.findIndex(i => i.id === id);
if (idx > -1) allImages.splice(idx, 1);
countersPestana[tab-1]--;
document.getElementById(`count${pestana}`).textContent = countersPestana[pestana-1];
updateStatistics();
}
}
function downloadIndividual(id) {
const img = allImages.find(i => i.id === id);
if (img) {
const a = document.createElement('a');
a.href = img.data;
a.download = `${img.codigoLlave}_${img.nombreCompleto}`;
a.click();
}
}
function exportKeys() {
const text = Array.from(codekeys).join('\n');
const blob = new Blob([text], { type: 'text/plain' });
const url = URL.createObjectURL(blob);
const a = document.createElement('a');
a.href = url;
a.download = `KEYS_${new Date().toISOString().slice(0,10)}.txt`;
a.click();
```

```
}

function showAlert(message) {

const alert = document.createElement('div');

alert.className = 'repeating-alert';

alert.innerHTML = `🚫 ${message}<br><button onclick="this.parentElement.remove()" style="margin-top:15px;padding:12px 25px;background:#10b981;color:white;border:none;border-radius:20px;font-weight:bold;cursor:pointer;">OK</button>`;

document.body.appendChild(alert);

setTimeout(() => alert.remove(), 5000);

}

function cleanIndexedDB() {

if (confirm('REMOVE EVERYTHING from IndexedDB PERMANENTLY?')) {

indexedDB.deleteDatabase('MitochondriaDB_Pro');

location.reload();

}

}

// EVENTS

window.onclick = function(event) {

if (event.target.id === 'modalHistoria') cerrarHistoriaClinica();

};


window.addEventListener('load', () => {

initIndexedDB();

document.getElementById('fechaConsulta').valueAsDate = new Date();

});


// 💾 AUTO-SAVE ON CLOSE

window.addEventListener('beforeunload', async () => {

if (dbIniciada && db) {

console.log('🗄️ AUTO-SAVED before closing...');

saveImagesDB();

saveHistoriesDB();

}

});

</script>

</body>

</html>
```
